# Exploring the low photosynthetic efficiency of cyanobacteria in blue light using a mutant lacking phycobilisomes

**DOI:** 10.1007/s11120-019-00630-z

**Published:** 2019-02-28

**Authors:** Veerle M. Luimstra, J. Merijn Schuurmans, Carolina F. M. de Carvalho, Hans C. P. Matthijs, Klaas J. Hellingwerf, Jef Huisman

**Affiliations:** 1 Department of Freshwater and Marine Ecology, Institute for Biodiversity and Ecosystem Dynamics, University of Amsterdam, PO Box 94240, 1090 GE Amsterdam, The Netherlands; 2 Wetsus, European Centre of Excellence for Sustainable Water Technology, Oostergoweg 9, 8911 MA Leeuwarden, The Netherlands; 3 Swammerdam Institute for Life Sciences, University of Amsterdam, PO Box 94248, 1090 GE Amsterdam, The Netherlands

**Keywords:** Blue light, Photosynthesis, Phycobilisomes, *Synechocystis* sp. PCC 6803, PAL mutant, Oxygen production

## Abstract

The ubiquitous chlorophyll *a* (Chl *a*) pigment absorbs both blue and red light. Yet, in contrast to green algae and higher plants, most cyanobacteria have much lower photosynthetic rates in blue than in red light. A plausible but not yet well-supported hypothesis is that blue light results in limited energy transfer to photosystem II (PSII), because cyanobacteria invest most Chl *a* in photosystem I (PSI), whereas their phycobilisomes (PBS) are mostly associated with PSII but do not absorb blue photons. In this paper, we compare the photosynthetic performance in blue and orange-red light of wildtype *Synechocystis* sp. PCC 6803 and a PBS-deficient mutant. Our results show that the wildtype had much lower biomass, Chl *a* content, PSI:PSII ratio and O_2_ production rate per PSII in blue light than in orange-red light, whereas the PBS-deficient mutant had a low biomass, Chl *a* content, PSI:PSII ratio, and O_2_ production rate per PSII in both light colors. More specifically, the wildtype displayed a similar low photosynthetic efficiency in blue light as the PBS-deficient mutant in both light colors. Our results demonstrate that the absorption of light energy by PBS and subsequent transfer to PSII are crucial for efficient photosynthesis in cyanobacteria, which may explain both the low photosynthetic efficiency of PBS-containing cyanobacteria and the evolutionary success of chlorophyll-based light-harvesting antennae in environments dominated by blue light.

## Introduction

Cyanobacteria are the oldest known oxygen-producing photosynthetic organisms on our planet and their photosynthetic activity is widely held responsible for oxygenation of the Earth’s atmosphere about 2.3 billion years ago (Holland 2002; Schirrmeister et al. 2015). The ubiquitous chlorophyll *a* (Chl *a*) pigment in the photosystems of oxygenic phototrophs absorbs both blue (440 nm) and red light (678 nm), and hence one would expect that these two colors of light are used at approximately equal efficiency. Yet, in contrast to green algae and higher plants, cyanobacteria appear to have much lower oxygen (O_2_) production and growth rates in blue light than in red light (e.g., Lemasson et al. 1973; Pulich and van Baalen 1974; Wilde et al. 1997; Tyystjärvi et al. 2002; Wang et al. 2007; Singh et al. 2009; Luimstra et al. 2018).

Why cyanobacteria perform less well in blue light is not yet fully resolved. It is often hypothesized that the distribution of absorbed light energy between photosystem I (PSI) and photosystem II (PSII) plays a key role (Fujita 1997; El Bissati and Kirilovsky 2001; Wang et al. 2007; Singh et al. 2009; Solhaug et al. 2014; Kirilovsky 2015; Luimstra et al. 2018). To optimize absorption of light energy, cyanobacteria use phycobilisomes (PBS) as light-harvesting antennae, which transfer absorbed light energy to the photosystems (Grossman et al. 1993; Tandeau de Marsac 2003). Most of the PBS are typically associated with PSII (state 1), but PBS can be relocated to PSI (state 2) at time scales of seconds to minutes, or they can be detached from the photosystems in which case they do not contribute to the photosynthetic activity of the cells (Joshua et al. 2005; Mullineaux 2014; Kirilovsky 2015). Blue light ≤ 450 nm is very poorly absorbed by PBS (Tandeau de Marsac 2003; Six et al. 2007), however, and hence PBS cannot distribute the absorbed light energy over the two photosystems.

Instead, blue light is directly absorbed by chlorophyll and carotenoids associated with the two photosystems. Cyanobacteria contain ~ 100 molecules of Chl *a* per PSI (Jordan et al. 2001) but only ~ 35 molecules of Chl *a* per PSII (Umena et al. 2011). Moreover, the PSI:PSII ratio of cyanobacteria usually ranges between 5:1 and 2:1, depending on the growth conditions, which is higher than the approximately 1:1 ratio often found in eukaryotic phototrophs (Shen et al. 1993; Fujita 1997; Olive et al. 1997; Singh et al. 2009; Allahverdiyeva et al. 2014). Since cyanobacteria usually invest most of their Chl *a* in PSI (e.g., Myers et al. 1980; Fujita 1997; Luimstra et al. 2018) and, in cyanobacteria, only the carotenoids in PSI are involved in light harvesting (Stamatakis et al. 2014), PSI will absorb more blue photons than PSII. This implies that blue light is likely to cause an excitation imbalance between both photosystems, with low O_2_ production by PSII and limited linear electron transport (Fujita 1997; Solhaug et al. 2014; Kirilovsky 2015), which then explains the low photosynthetic efficiency and growth rate of cyanobacteria in blue light (Luimstra et al. 2018). However, although many observations support this hypothesis, conclusive evidence is still limited.

In this paper, we aim to contribute to a further understanding of why cyanobacteria display a low photosynthetic efficiency in blue light. Therefore, we compare the photosynthetic efficiency in blue and orange-red light of *Synechocystis* sp. PCC 6803 and a mutant strain lacking PBS known as the PAL mutant (Ajlani and Vernotte 1998). The PAL mutant does not have light-harvesting antennae that are able to redistribute light energy over both photosystems. Consequently, our expectation is that the PAL mutant will have limited energy transfer to PSII irrespective of the light color and hence will display a similarly low photosynthetic efficiency in both orange-red and blue light as the wildtype in blue light. An advanced understanding of the light-color dependence of cyanobacterial photosynthesis may contribute to an improved design of successful culture conditions in biotechnological applications, and to further clarification of the ecological distributions of cyanobacteria in waters dominated by different wavelengths.

## Materials and methods

### Strains and culture conditions

We investigated a wildtype and a mutant strain of *Synechocystis* sp. PCC 6803, which uses the phycobilin-pigment C-phycocyanin in its PBS. The wildtype strain was provided by professor D. Bhaya (University of Stanford, USA) and the phycobilisome-deficient PAL mutant strain was provided by Dr. G. Ajlani (Université Paris-Sud, France).

**Fig. 1:**
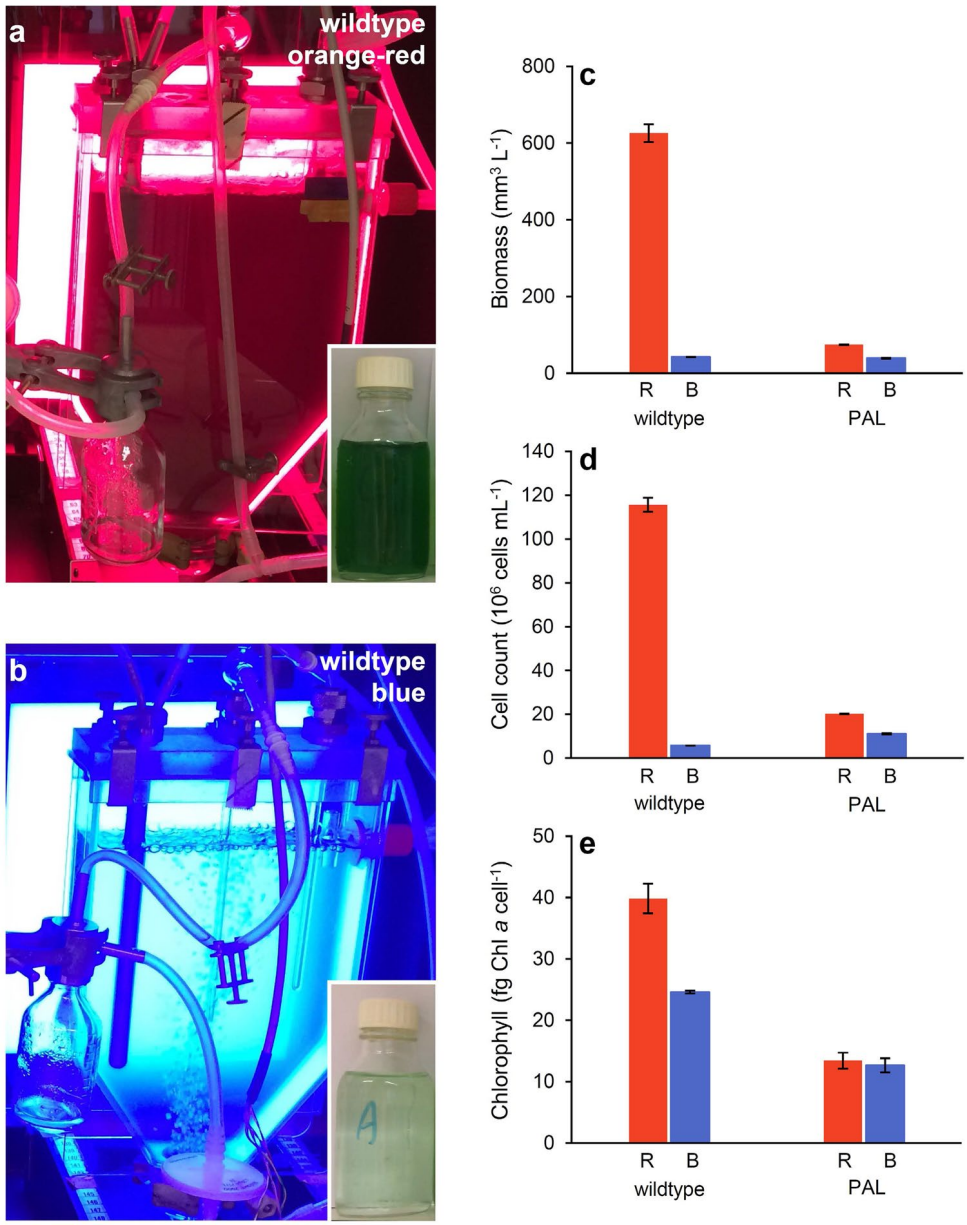
*Synechocystis* sp. PCC 6803 wildtype and the PAL mutant were grown in light-limited chemostats and provided with either **a** orange-red (660 nm) or **b** blue (450 nm) LED light. Insets show samples taken from steady-state chemostats of the wildtype, which illustrate that the wildtype produced much higher biomass in orange-red than in blue light. Bar graphs show biomass (**c**), cell counts (**d**) and cellular Chl *a* content (**e**) of each strain at steady state in orange-red (R) and blue (B) light. Biomass is expressed as total biovolume of the cells per L. Data show the averages of three (biomass and cell counts) or two (Chl *a* content) technical replicates ± SD

The experimental set-up is illustrated in Fig. 1a,b. The strains were grown at 30 °C in 1.8 L light-limited chemostats with flat culture vessels illuminated from one side (Huisman et al. 2002). Blue light (450 nm) or orange-red light (660 nm) with a full width at half maximum of ~ 20 nm was provided by narrow-band LED panels (Philips Lighting B.V., Eindhoven, The Netherlands) at an incident light intensity of 45 µmol photons m^−2^ s^−1^. Light intensities were measured with an LI-250 light meter (LI-COR Biosciences, Lincoln, NE, USA). The chemostats had an optical path length of 5 cm. Cultures were provided with BG-11 medium (Merck, Darmstadt, Germany) supplemented with 5 mM Na_2_CO_3_, at a constant dilution rate of D = 0.015 h^−1^ (0.36 day^−1^). Chloramphenicol (20 µg mL^−1^) was added to the first 6 L of medium for the PAL mutant to prevent growth of the wildtype (Ajlani and Vernotte 1998). The cultures were mixed by bubbling with CO_2_-enriched air (2% v/v) flowing at a rate of 30 L h^−1^. The CO_2_ concentration of the gas mixture was regularly monitored using an Environmental Gas Monitor for CO_2_ (EGM-4; PP Systems, Amesbury, MA, USA).

### Cell counts and chlorophyll analysis

A CASY 1 TTC cell counter with a 60-µm capillary (Schärfe Systems GmbH, Reutlingen, Germany) was used to count cells and measure their biovolume. Samples were diluted to ~ 5×10^4^ cells mL^−1^ in Casyton solution prior to measuring. Chl *a* content of the cells was measured spectrophotometrically using a Novaspec III Visible Spectrophotometer after extraction in 80% (v/v) acetone/5% (v/v) DMSO (Porra et al. 1989).

### Absorption and 77 K fluorescence spectra

After the chemostats reached steady state, 1 mL and 1.5 mL samples were taken for the measurement of light absorption spectra and 77 K fluorescence spectra, respectively. Immediately after sampling, light absorption spectra were measured from 400 to 750 nm using an updated Aminco DW2000 spectrophotometer (OLIS, Bogart, GA, USA). The spectra were normalized to Chl *a* absorbance at 678 nm, after baseline correction for minimum absorbance at 750 nm. Molar phycocyanin-to-Chl *a* (PC:Chl) ratios were estimated from the absorption spectra according to Rakhimberdieva et al. (2001):

$${\text{PC}}:{\text{Chl}}=~\frac{{4.9{A_{625}}~-~2.1{A_{652}}~-~0.8{A_{678}}}}{{0.1{A_{625}}~-~0.7{A_{652}}~+~15.8{A_{678}}}},$$

where *A*_625_, *A*_652_, and *A*_678_ are the absorption peaks of PC, allophycocyanin, and Chl *a*, respectively. Cellular PC contents (quantified as monomers) were calculated from the measured Chl *a* content and estimated PC:Chl ratio.

For the 77 K fluorescence spectra, the 1.5 mL samples were transferred to 3-mL cuvettes prefilled with 1.5 mL 60% glycerol. The final glycerol concentration in the cuvettes was 30% to minimize dissociation of PBS, and cell concentrations were below 4 × 10^7^ cells mL^−1^ to minimize re-absorption of the fluorescence signal (Mao et al. 2003). Samples were mixed by pipetting up and down three times and the cuvettes were frozen in liquid nitrogen. To guarantee that the fluorescence spectra reflected the quantities of photosynthetic components at the time of sampling, this procedure was performed within 20 s. Cuvettes were stored at − 80 °C, for maximally 1 week, until 77 K fluorescence analysis with an OLIS DM45 spectrofluorimeter (OLIS, Bogart, GA, USA) equipped with a Dewar cell. Fluorescence emission was measured from 630 to 750 nm with excitation at 440 nm (mainly Chl *a*). The peak areas of PSI (emission at 725 nm) and PSII (emission at 695 nm) were calculated by deconvolution of the spectra according to Du et al. (2016) using R version 3.3.3 (R Development Core Team 2017).

Earlier studies have shown that the ratio of the surface areas of the PSI and PSII peaks in the 77 K fluorescence emission spectra corresponds quite well to the molar PSI:PSII ratio of the actual protein complexes (Murakami 1997; Schuurmans et al. 2017). Therefore, cellular PSI and PSII contents (in mol cell^−1^) were calculated using the PSI:PSII ratio obtained from the deconvoluted 77 K spectra and the cellular Chl *a* content (mol cell^−1^, where Chl *a* has a molecular weight of 893.5 g mol^−1^). The calculation assumes that PSI and PSII contain 100 and 35 Chl *a* molecules, respectively (Jordan et al. 2001; Umena et al. 2011):

$${\text{PS}}{{\text{I}}_{{\text{cell}}}}=~\frac{{\left[ {{\text{Chl}}~a} \right]~}}{{100+35/({\text{PSI}}\!\!:\!\!{\text{PSII}})}}$$

$${\text{PSI}}{{\text{I}}_{{\text{cell}}}}=~\frac{{\left[ {{\text{Chl~}}a} \right]~}}{{100\left( {{\text{PSI}}\!\!:\!\!{\text{PSII}}} \right)+35}}.$$

We note that these calculations should be interpreted with care, and provide only a rough estimate of relative changes in PSI and PSII content.

### Oxygen production and consumption rates analyzed using MIMS

O_2_ production and consumption were measured using membrane-inlet mass spectrometry (MIMS), via high-vacuum-supported diffusive equilibration of the dissolved O_2_ through a gas permeable membrane coupled to a mass spectrometer (Kana et al. 1994). Small amounts of gasses can flow out of the liquid culture into the sensor of the mass spectrometer through a thin Teflon membrane. Addition of ^18^O_2_ and subsequent quantification of both stable oxygen isotopes (^16^O_2_ and ^18^O_2_) allows for distinction between O_2_ production (evolution of ^16^O_2_ from water-splitting at PSII) and O_2_ consumption (uptake of the added ^18^O_2_) in a single sample. Samples were taken from the steady-state continuous cultures of *Synechocystis* sp. PCC 6803 wildtype and the PAL mutant acclimated to orange-red and blue light. To facilitate comparison, all samples were diluted to the same OD_750_ of 0.04 prior to the MIMS analysis.

A fresh sample was taken for each measurement and transferred to a double-walled, airtight 10-mL glass chamber (a DW3 cuvette from Hansatech Instruments, modified by the Technology Centre of the University of Amsterdam) equipped with a magnetic stirrer. The inlet sensor of the mass spectrometer was placed directly in the culture. Prior to measurements, NaHCO_3_ was added at a final concentration of 15 mM to prevent carbon limitation during the experiment. The samples were flushed with N_2_ gas to reduce the ^16^O_2_ concentration to ~ 5% of the value in air-equilibrated medium to prevent O_2_ saturation during the experiment, after which ^18^O_2_ (95–98% pure; Cambridge Isotope Laboratories) was added in the headspace. The magnetic stirrer allowed for diffusion into the liquid culture. When the ^18^O_2_ concentration reached ~ 20% of the value in air-equilibrated medium, the headspace was aspirated and the glass chamber closed.

Temperature in the glass chamber was maintained at 30 °C by water pumped through its double wall. Two LED lamps, identical to those used for the chemostats, illuminated the glass chamber from both sides to minimize shading effects. The samples were exposed to orange-red or blue light at increasing light intensities (summed over both lamps) from 0 to 400 µmol photons m^−2^ s^−1^. After 10-min dark adaptation, light intensity was increased every 3 min, while O_2_ concentrations were measured continuously by the MIMS. The MIMS signals were normalized to argon according to Kana et al. (1994), and O_2_ production and consumption rates were calculated according to Bañares-España et al. (2013).

O_2_ production and consumption rates were expressed as fmol O_2_ per cell per minute and as mol O_2_ per mol PSII per second. Cell densities were determined directly after each MIMS experiment. Gross O_2_ production (*P*) rates were plotted versus light intensity (*I*) and fitted to *P*–*I* curves using a hyperbolic tangent function (Platt and Jassby 1976):

$$P={P_{\hbox{max} ~}}\tanh \left( {\frac{{\alpha ~I}}{{{P_{\hbox{max} }}}}} \right),$$

where *P*_max_ is the maximal rate of O_2_ production and α is the affinity for light (i.e., the initial slope of the *P*–*I* curve). The fits were based on a nonlinear least-squares regression using R version 3.3.3 (R Development Core Team 2017).

## Results

### The PAL mutant produced less biomass in both orange-red and blue light

Photos of the steady-state chemostats illustrate the conspicuous difference in growth performance between *Synechocystis* sp. PCC 6803 wildtype grown in orange-red (Fig. 1a) and in blue light (Fig. 1b). Both strains grew sufficiently well to sustain a steady state, which implies that their maximum specific growth rates exceeded the dilution rate of 0.015 h^−1^ in both light colors. However, the wildtype produced much higher steady-state biomass and cell numbers in orange-red light than in blue light (Fig. 1c, d). By contrast, the steady-state biomass and cell numbers of the PAL mutant were low in both orange-red and blue light, and comparable to those of the wildtype in blue light (Fig. 1c, d).

Furthermore, the wildtype had a higher Chl *a* content per cell in orange-red light than in blue light, whereas the PAL mutant had a low Chl *a* content in both orange-red and blue light (Fig. 1e). The wildtype had a lower cell volume in orange-red light (5.4 ± 0.03 µm^3^) than in blue light (7.4 ± 0.03 µm^3^), whereas the PAL mutant produced smaller cells in both light colors (3.6 ± 0.1 µm^3^).

### The PAL mutant showed identical absorption spectra in both orange-red and blue light

**Fig. 2:**
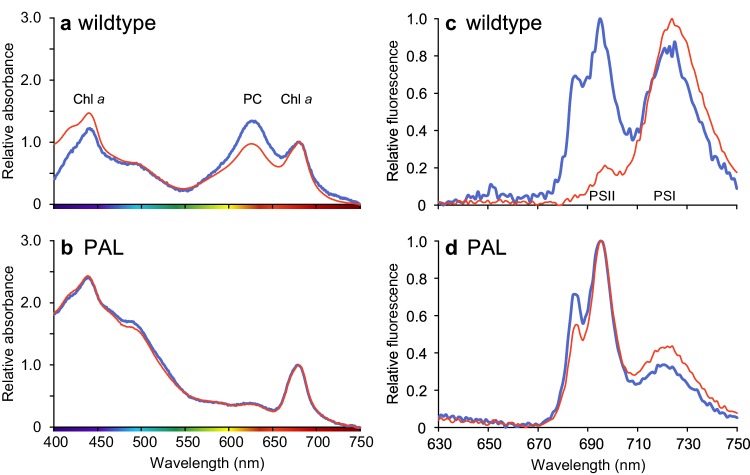
(**a, b**) Light absorption spectra of *Synechocystis* sp. PCC 6803 wildtype (**a**) and PAL mutant (**b**) sampled from steady-state chemostats acclimated to either orange-red (red line) or blue (blue line) light. Main absorption peaks of chlorophyll *a* (Chl *a*) and phycocyanin (PC) are indicated in panel **a**. Light absorption was normalized to Chl *a* absorbance at 678 nm, after baseline correction for minimum absorbance at 750 nm. **c, d** Low-temperature (77 K) fluorescence emission spectra of *Synechocystis* sp. PCC 6803 wildtype (**c**) and PAL mutant (**d**). Excitation of the cells at 440 nm (mainly Chl *a*) yields fluorescence emission peaks around 695 nm for PSII and around 720 nm for PSI, as indicated in panel **c**. The 77 K fluorescence emission spectra were normalized to the minimum and maximum emission of each spectrum. All spectra show the averages of three technical replicates

Absorption spectra normalized to Chl *a* revealed that *Synechocystis* sp. PCC 6803 wildtype had lower PC:Chl ratios in orange-red light than in blue light (Fig. 2a; Table 1). By contrast, the PAL mutant lacks absorption by PC (at 625 nm) and its absorption spectra were completely identical when grown in orange-red and blue light (Fig. 2b). Furthermore, the PAL mutant had a bleached appearance with a low Chl *a* content per cell and showed higher absorption in the blue part of the spectrum, both when acclimated to blue and when acclimated to orange-red light (Fig. 2b).

**Table 1:** Cellular contents of Chl *a*, PC, PSI and PSII of the wildtype and PAL mutant, in steady-state chemostats acclimated to either orange-red or blue light

	Wildtype		PAL mutant		Units
	Orange-red	Blue	Orange-red	Blue	
Chl *a* content^a^	45	28	15	14	10^−18^ mol cell^−1^
PSI content^b^	0.43	0.22	0.11	0.09	10^−18^ mol cell^−1^
PSII content^b^	0.04	0.15	0.12	0.14	10^−18^ mol cell^−1^
PSI:PSII ratio^c^	11.7	1.5	0.9	0.7	–
PC content^d^	8.0	7.4	n.a.	n.a.	10^−18^ mol cell^−1^
PC:Chl ratio^e^	0.18	0.26	n.a.	n.a.	–
PC:PSII ratio^f^	217	50	n.a.	n.a.	–

^a^Chl *a* content was measured spectrophotometrically

^b^PSI and PSII content were calculated from the Chl *a* content and PSI:PSII ratio

^c^PSI:PSII ratio was estimated from deconvolution of the 77 K fluorescence spectra

^d^PC content was calculated from the Chl *a* content and PC:Chl ratio

^e^PC:Chl ratio was estimated from the absorption spectra

^f^PC:PSII ratio was calculated from the PC and PSII content

### The PAL mutant had a low PSI:PSII ratio in both orange-red and blue light

Fluorescence peaks of PSI and PSII at 725 nm and 695 nm, respectively, were very prominent in the 77 K fluorescence spectra (Fig. 2c, d). The wildtype allocated most of its Chl *a* to PSI when grown in orange-red light, whereas it decreased its allocation to PSI and increased its allocation to PSII when grown in blue light (Fig. 2c). Hence, the wildtype had much lower PSI:PSII ratios and PC:PSII ratios in blue light than in orange-red light (Table 1). By contrast, the PAL mutant had a relatively low PSI content and high PSII content in both light colors, resulting in a low PSI:PSII ratio in both orange-red and blue light (Fig. 2d; Table 1).

The blue-light acclimated wildtype showed an additional fluorescence peak at 685 nm (Fig. 2c), which is commonly attributed to the chlorophyll antenna CP43 in PSII (Andrizhiyevskaya et al. 2005; Wilson et al. 2007; Brecht et al. 2014). In the PAL mutant, this peak was present in both orange-red and blue light (Fig. 2d).

### The PAL mutant had a low oxygen production in both orange-red and blue light

**Fig. 3:**
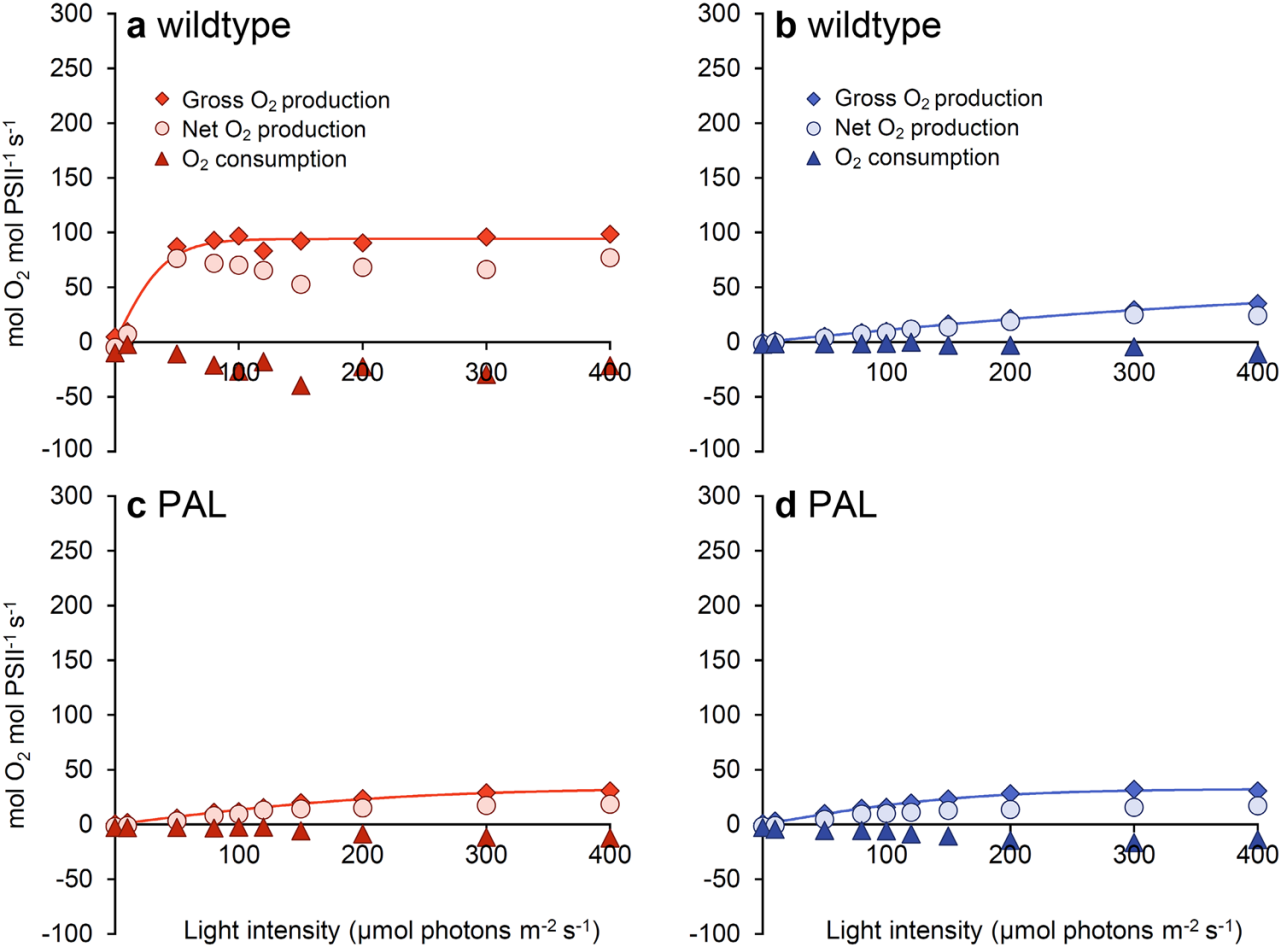
O_2_ production and consumption rates per PSII of *Synechocystis* sp. PCC 6803 wildtype (**a, b**) and the PAL mutant (**c, d**). Cells were acclimated to orange-red (**a, c**) or blue (**b, d**) light and subsequently exposed to increasing intensities of the same light color. The data show averages of gross photosynthetic O_2_ production (diamonds), net O_2_ production (circles), and O_2_ consumption (triangles), measured in duplicate samples using membrane-inlet mass spectrometry (MIMS). Lines represent *P*–*I* curves fitted to the gross O_2_ production using the hyperbolic tangent function; parameter estimates are given in Table 2

**Fig. 4:**
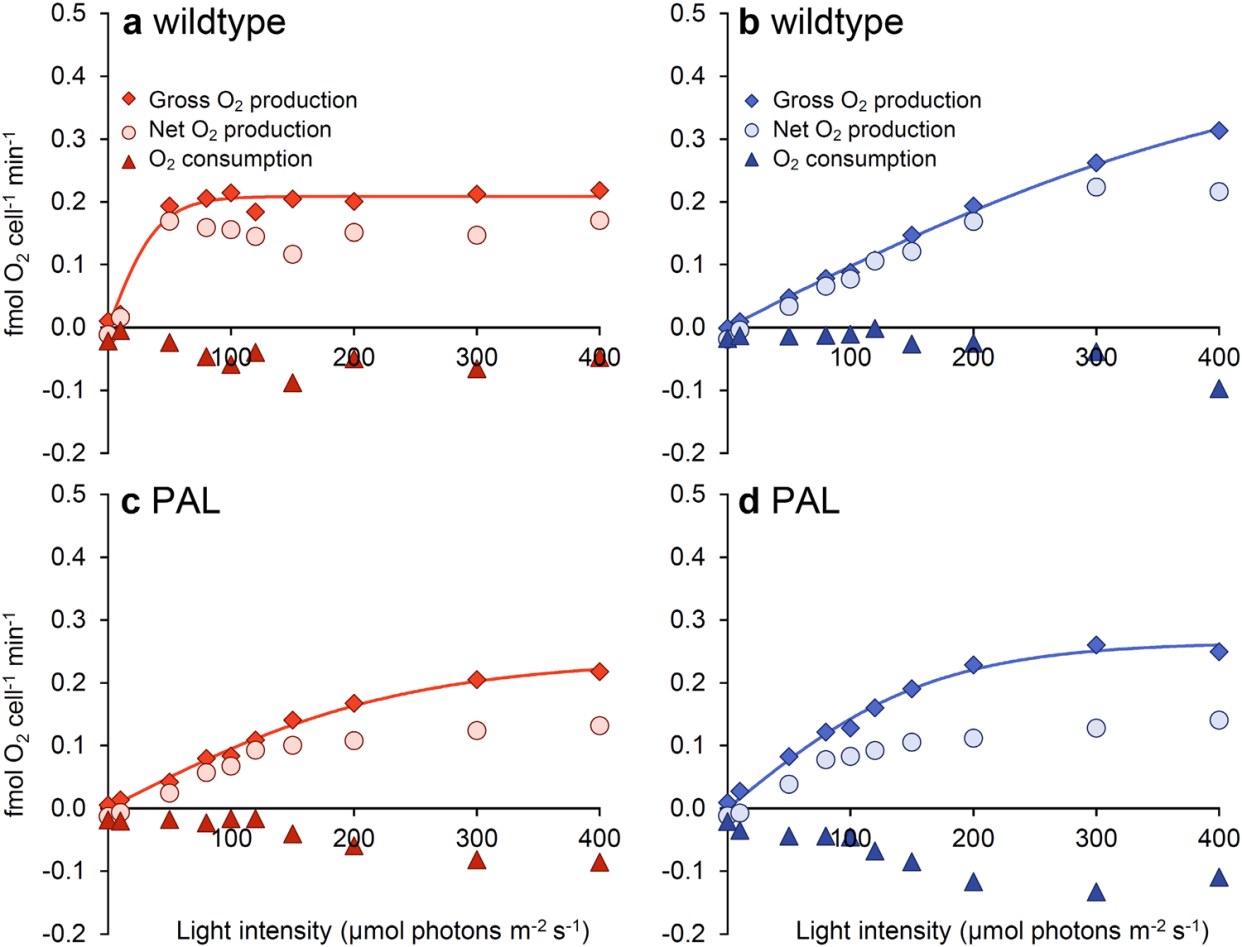
O_2_ production and consumption rates per cell of *Synechocystis* sp. PCC 6803 wildtype (**a, b**) and the PAL mutant (**c, d**). See Fig. 3 for further details

Membrane-inlet mass spectrometry (MIMS) was used to separately measure photosynthetic O_2_ production and O_2_ consumption at different light intensities. O_2_ production and consumption rates were expressed in two different ways: per mol PSII (Fig. 3) and per cell (Fig. 4). We note that our estimates of the O_2_ production rate per PSII are consistent with turnover rates of the PSII-water oxidizing complex measured in microalgae (Ananyev and Dismukes 2005).

**Table 2:** Photosynthetic parameters estimated by fitting a hyperbolic tangent to the gross O_2_ production rate per PSII and the gross O_2_ production rate per cell. Standard errors are indicated by bracketed values

Parameter	Wildtype		PAL mutant	
	Orange-red	Blue	Orange-red	Blue
O_2_ production per PSII (mol O_2_ mol PSII^−1^ s^−1^)				
*α*	2.32 (0.43)	0.11 (0.00)	0.14 (0.01)	0.20 (0.01)
*P*_max_	94.2 (3.1)	50.1 (3.5)	33.6 (1.3)	32.6 (1.0)
O_2_ production per cell (fmol O_2_ cell^−1^ min^−1^)				
*α*	5.1 × 10^−3^ (9.6 × 10^−4^)	1.0 × 10^−3^ (0.3 × 10^−4^)	1.0 × 10^−3^ (0.4 × 10^−4^)	1.6 × 10^−3^ (0.7 × 10^−4^)
*P*_max_	0.209 (0.007)	0.446 (0.032)	0.237 (0.009)	0.265 (0.008)

In the wildtype, net and gross O_2_ production per PSII increased much more steeply with light intensity (had higher α) and maximum O_2_ production rates per PSII (higher *P*_max_) were higher in orange-red than in blue light (Fig. 3a, b; Table 2). By contrast, the PAL mutant had low α and low *P*_max_ in both orange-red and blue light (Fig. 3c, d), very similar to the low α and low *P*_max_ of the wildtype in blue light (Fig. 3b).

When O_2_ production and consumption rates were expressed per cell rather than per PSII, we obtained similar results (Fig. 4). The wildtype again had higher α in orange-red light than in blue light (Fig. 4a, b; Table 2), whereas the PAL mutant had a low α in both orange-red and blue light comparable to the low α of the other strains in blue light (Fig. 4c, d). However, in contrast to *P*_max_ per PSII, *P*_max_ per cell was of similar magnitude in blue and orange-red light for both strains (Fig. 4).

## Discussion

### What happens if phycobilisomes cannot be used?

Our results show that *Synechocystis* sp. PCC 6803 wildtype displays much lower O_2_ and biomass production rates in blue light than in orange-red light, in agreement with previous studies (e.g., Wilde et al. 1997; El Bissati and Kirilovsky 2001; Tyystjärvi et al. 2002; Singh et al. 2009; Bland and Angenent 2016; Luimstra et al. 2018). We tested the hypothesis that cyanobacteria have a low photosynthetic efficiency in blue light because PBS do not absorb wavelengths ≤ 450 nm, and hence blue light mainly excites PSI while fewer photons excite the PSII reaction center (Myers et al. 1980; Fujita 1997; Solhaug et al. 2014; Kirilovsky 2015). This results in an excitation imbalance between the two photosystems in blue light (Luimstra et al. 2018). The PAL mutant lacks PBS. Therefore, if our hypothesis is correct, then the PAL mutant should have (i) a similar low biomass and O_2_ production rate and (ii) a similar low PSI:PSII ratio in both orange-red and blue light as the wildtype in blue light. This reasoning is confirmed by our experiments, in which the PAL mutant indeed displayed a similar low biomass (Fig. 1c), cell production (Fig. 1d), Chl *a* content (Fig. 1e), PSI:PSII ratio (Fig. 2c,d; Table 1), and O_2_ production rate per PSII (Fig. 3) in both orange-red and blue light as the wildtype in blue light. In other words, the poor photosynthetic performance of PBS-containing cyanobacteria in blue light was similar to the performance of a PBS-deficient mutant in both light colors.

We note that our results also show some differences between the wildtype in blue light and the PAL mutant. In particular, the photophysiology of the PAL mutant shows enhanced absorption in the 400–500 nm range (Fig. 2b), indicative of enhanced carotenoid production (see also Ajlani and Vernotte 1998; Kwon et al. 2013) and an even lower PSI:PSII ratio than the wildtype in blue light (Fig. 2d). Hence, in a sense, the PAL mutant lacking PBS might be interpreted as a more extreme phenotype in comparison to the wildtype that contains PBS but cannot use them in blue light.

The low growth rate and low PSI:PSII ratio of the PAL mutant have also been described by many other studies (Ajlani and Vernotte 1998; El Bissati and Kirilovsky 2001; Bernát et al. 2009; Stadnichuk et al. 2009; Collins et al. 2012; Kwon et al. 2013; Liberton et al. 2017). Comparison of different antenna mutants revealed that the PSI:PSII ratio decreases with a decrease in antenna size of the PBS, as a response to the diminished energy transfer from PBS to PSII when the antenna size is gradually reduced (Ajlani et al. 1995; Olive et al. 1997; Bernát et al. 2009; Stadnichuk et al. 2009; Collins et al. 2012; Kwon et al. 2013; Liberton et al. 2017). Likewise, Page et al. (2012) reported that growth rates were highest in the *Synechocystis* PCC 6803 wildtype, and decreased progressively with a decrease in antenna size of a series of antenna mutants. We also note the presence of the fluorescence peak at 685 nm in the 77 K fluorescence spectra of the PAL mutant (Fig. 2d), which has also been found in previously published 77 K spectra of the PAL mutant (Ajlani and Vernotte 1998; Bernát et al. 2009; Stadnichuk et al. 2009; Collins et al. 2012; Kwon et al. 2013). This peak, which is commonly attributed to the chlorophyll-binding protein CP43 in PSII (Andrizhiyevskaya et al. 2005; Wilson et al. 2007; Brecht et al. 2014), is also present in the wildtype in blue light (Fig. 2c). Hence, our findings with the PAL mutant are well in line with many previous studies. The novelty of our results is the recognition that, in many respects, the phenotype of the PAL mutant resembles the phenotype of the wildtype strain in blue light. This is further confirmed by our analysis of the oxygen production rates of the wildtype and PAL mutant, as measured by MIMS (Figs. 3, 4).

It has been shown that deletion of PBS in the PAL mutant is accompanied by profound changes in the proteome in comparison to the wildtype (Kwon et al. 2013; Liberton et al. 2017). Kwon and colleagues report a major increase of PSII proteins accompanied by a slight decrease of PSI proteins in the PAL mutant. Furthermore, they found that the PAL mutant has increased abundances of proteins involved in stress responses to high light and in carbohydrate metabolism (mostly gluconeogenesis). More recent proteome analyses by Liberton et al. (2017) support these findings, and in addition these authors found a decrease in bicarbonate transport proteins and an increase in transport proteins involved in nitrate uptake in the PAL mutant. Interestingly, the physiology of the wildtype is also completely changed when transferred from white to blue light (Singh et al. 2009). Transcriptome analysis by Singh et al. revealed that *Synechocystis* sp. PCC 6803 wildtype cells grown in blue light showed, among others, an increased expression of genes encoding PSII subunits and proteins involved in stress responses to high light, carbohydrate metabolism, and nitrate uptake, which resembles several of the proteome results in the PAL mutant (Kwon et al. 2013; Liberton et al. 2017). This indicates again that the PAL mutant and wildtype in blue light share many similarities, not only in their photophysiological traits but also in terms of their cellular metabolism.

### Implications for biotechnology

Several studies have argued that truncation of light-harvesting antennae may be an advantage in crop and algal biomass production for, e.g., biotechnological applications (see, e.g., Ort and Melis 2011; Work et al. 2012; de Mooij et al. 2015; Kirst et al. 2017). In dense algal cultures or plant canopies, cells near the surface absorb excessive amounts of light energy that they largely dissipate as heat, while cells deeper down in the culture receive insufficient light for photosynthesis (Melis 2009; Blankenship and Chen 2013). As a consequence, much of the absorbed light energy is wasted rather than invested in biomass production. Truncation of the light-harvesting antennae reduces absorption per individual cell and thereby distributes light more evenly among the cells in dense algal cultures. It has indeed been demonstrated that antenna truncation can increase the biomass production of green algae (e.g., Polle et al. 2002; Perrine et al. 2012; de Mooij et al. 2015; Shin et al. 2016).

Our results indicate that truncation of the PBS of cyanobacteria has other effects than the truncation of the chlorophyll-based light-harvesting complexes of green algae. Green algae generally maintain a PSI:PSII ratio of around 1:1, in contrast to cyanobacteria which usually have a much higher PSI:PSII ratio (Shen et al. 1993; Singh et al. 2009; Kirilovsky 2015) and mainly use their PBS to direct sufficient light energy to PSII. Therefore, truncation of PBS in cyanobacteria will have a stronger effect on light redistribution between the two photosystems than truncation of the light-harvesting complex in green algae. In particular, our findings show that complete deletion of the PBS of cyanobacteria, in the PAL mutant, yields an excitation imbalance between the two photosystems, which results in a low PSII activity (Fig. 3) and low biomass production (Fig. 1) in both blue and orange-red light. This matches earlier results of, e.g., Ajlani and Vernotte (1998), Bernát et al. (2009), Page et al. (2012), and Kwon et al. (2013), who also found that the PAL mutant has a lower biomass production than the wildtype.

Possibly, partial truncation of the antennae by shortening or removing the rods of the PBS might improve the biomass production of cyanobacteria, as the PBS can then still distribute the absorbed light energy over the two photosystems while overall absorbance per cell is lower (Lea-Smith et al. 2014). Partial truncation of the PBS likely implies that less light will be transferred to PSII, which must be compensated by a decreased PSI:PSII ratio, to maintain the balance between the two photosystems. Several cyanobacterial studies show that partial truncation mutants indeed decreased their PSI:PSII ratio progressively and proportionally to a decrease in antenna size (Ajlani et al. 1995; Olive et al. 1997; Bernát et al. 2009; Stadnichuk et al. 2009; Collins et al. 2012; Kwon et al. 2013; Liberton et al. 2017). Although most of these mutants did not show a higher productivity than the wildtype, removal of the phycocyanin rods but not the allophycocyanin core in the Olive mutant (Rögner et al. 1990) resulted in an enhanced cyanobacterial productivity at high light intensities (Kwon et al. 2013) and low-carbon conditions (Lea-Smith et al. 2014).

### Implications for cyanobacterial photosynthesis in the blue ocean

Marine cyanobacteria of the *Synechococcus* genus often use the phycobilin pigments phycoerythrobilin (PEB) and phycourobilin (PUB) to absorb wavelengths in the green part (525–575 nm) and blue-green part (475–515 nm) of the light spectrum (Six et al. 2007; Grébert et al. 2018). These phycobilin pigments do not absorb photons in the violet-blue part of the spectrum (≤ 450 nm), and hence, similar to *Synechocystis* PCC 6803, marine *Synechococcus* will be unable to use their PBS to harvest blue photons ≤ 450 nm for PSII (Luimstra et al. 2018). Consequently, *Synechococcus* usually thrives in the near-surface layers of the oceans and in coastal waters, where green and blue-green light is available (Partensky et al. 1999; Scanlan and West 2002; Stomp et al. 2007a).

Cyanobacteria of the genus *Prochlorococcus* abound in the subtropical ocean gyres and usually their populations extend deeper down in the water column than *Synechococcus* (Partensky et al. 1999; Scanlan and West 2002). They use a different light-harvesting strategy. *Prochlorococcus* does not deploy PBS, but has evolved light-harvesting antennae that effectively absorb blue light using divinyl-Chl *a* and *b* (Chisholm et al. 1992; Ting et al. 2002; Stomp et al. 2007b). *Prochlorococcus* likely evolved from a PBS-containing cyanobacterium (Kettler et al. 2007; Scanlan et al. 2009), and some *Prochlorococcus* strains still have genes for the synthesis of phycoerythrin (Hess et al. 1996). Interestingly, our results show that the loss of PBS does not have major negative fitness consequences in habitats dominated by blue light, as the PAL mutant had a similarly low biomass and cell production in blue light as the wildtype. Furthermore, we note that the 685-nm peak in our 77 K fluorescence spectra, indicative of the chlorophyll-binding protein CP43, was strongly induced by blue light (Fig. 2c) and in the PBS-deficient mutant (Fig. 2d; see also Ajlani and Vernotte 1998; Kwon et al. 2013). The chlorophyll-binding proteins (Pcb) in the light-harvesting antennae of *Prochlorococcus* are closely related to CP43 (Chen and Bibby 2005). Our results therefore suggest that the loss of PBS and transformation from an ancestral chlorophyll-binding protein of the CP43 family to Pcb caused a major evolutionary transition, from the poor photosynthetic performance in blue light by PBS-based cyanobacteria to efficient utilization of blue light by the chlorophyll-based light-harvesting antennae of *Prochlorococcus*. Harnessing of blue light will have provided a major selective advantage for *Prochlorococcus* over PBS-containing cyanobacteria in the deep blue waters of the open ocean.
